# Particle MCMC algorithms and architectures for accelerating inference in state-space models^☆^

**DOI:** 10.1016/j.ijar.2016.10.011

**Published:** 2017-04

**Authors:** Grigorios Mingas, Leonardo Bottolo, Christos-Savvas Bouganis

**Affiliations:** a Department of Electrical and Electronic Engineering, Imperial College London, London, SW7 2AZ, UK; b Department of Medical Genetics, University of Cambridge, Cambridge, CB2 0QQ, UK

**Keywords:** Markov Chain Monte Carlo, Particle filter, Field programmable gate array, Bayesian inference, Hardware acceleration

## Abstract

Particle Markov Chain Monte Carlo (pMCMC) is a stochastic algorithm designed to generate samples from a probability distribution, when the density of the distribution does not admit a closed form expression. pMCMC is most commonly used to sample from the Bayesian posterior distribution in State-Space Models (SSMs), a class of probabilistic models used in numerous scientific applications. Nevertheless, this task is prohibitive when dealing with complex SSMs with massive data, due to the high computational cost of pMCMC and its poor performance when the posterior exhibits multi-modality. This paper aims to address both issues by: 1) Proposing a novel pMCMC algorithm (denoted ppMCMC), which uses multiple Markov chains (instead of the one used by pMCMC) to improve sampling efficiency for multi-modal posteriors, 2) Introducing custom, parallel hardware architectures, which are tailored for pMCMC and ppMCMC. The architectures are implemented on Field Programmable Gate Arrays (FPGAs), a type of hardware accelerator with massive parallelization capabilities. The new algorithm and the two FPGA architectures are evaluated using a large-scale case study from genetics. Results indicate that ppMCMC achieves 1.96x higher sampling efficiency than pMCMC when using sequential CPU implementations. The FPGA architecture of pMCMC is 12.1x and 10.1x faster than state-of-the-art, parallel CPU and GPU implementations of pMCMC and up to 53x more energy efficient; the FPGA architecture of ppMCMC increases these speedups to 34.9x and 41.8x respectively and is 173x more power efficient, bringing previously intractable SSM-based data analyses within reach.

## Highlights

• Novel algorithmic and hardware techniques for fast SSM inference are proposed.
• New algorithm extends applicability of particle MCMC to multi-modal posteriors.
• FPGA architectures exploit particle and chain parallelism to accelerate sampling.
• 42x speedup vs. state-of-the-art CPU/GPU samplers is achieved for large problems.

## 1 Introduction

Markov Chain Monte Carlo (MCMC) algorithms are one of the fundamental tools used to sample from complex probability distributions. They are popular for sampling from posterior distributions in Bayesian inference, which are typically complex and have large dimensions. These samples are used to estimate integrals, which are needed in order to infer parameters, make predictions, perform model comparison, etc. [1]. This process is called Monte Carlo integration. [2] and [1] contain examples of how MCMC samples are used in various applications.

Most MCMC algorithms [1] are based on the assumption that the density of the sampled distribution (denoted p(θ), where *θ* is the sampled variable) can be evaluated pointwise up to multiplicative constant. Nevertheless, there are modeling scenarios in Bayesian statistics where it is not possible to evaluate the probability density because it does not admit a closed form expression. These include inference for State Space models (SSMs) [3], stochastic kinetic models [4], undirected graphical models [5]. These cases are often called “analytically intractable” in the MCMC literature.

The pseudo-marginal MCMC sampler introduced by [6] marked a major breakthrough in this field: It showed that if an unbiased estimator of the sampled density is used inside MCMC (instead of the closed form expression), the sampler still converges to the correct distribution. A large body of work has exploited this remarkable property during the last five years to construct new MCMC algorithms. The most widely applied among them is Particle MCMC (pMCMC) [3], which is designed for inference on SSMs with unknown parameters.

SSMs with unknown parameters is a widely used class of Bayesian models. They are applicable to problems where a sequence of unknown (hidden) states needs to be inferred from known observations and the relationships between states and observations have unknown parameters which also need to be inferred. A simple example is target tracking, where both the position of the tracked object and the noise in sensor measurements are unknown and need to be inferred from the measurements. This type of SSM inference is known to lead to analytically intractable densities [3]. pMCMC uses a Particle Filter (PF) [7] to unbiasedly estimate the density (which is a natural choice for SSMs). Therefore, it is capable of inferring the SSM posterior.

pMCMC has extended the applicability of SSMs to areas like ecology [8], communication networks [9], systems biology [10] and marine biogeochemistry [11]. Nevertheless, the computational cost of estimating the density using a PF is O(T⋅P) (where *T* is the number of hidden SSM states and *P* is the number of particles of the PF) [12]. Moreover, each pMCMC iteration requires a separate PF run and typically thousands of iterations are needed. This means that using pMCMC for large SSMs is computationally prohibitive. When conventional Central Processing Units (CPUs) are employed the runtimes can reach months or years [11], [10]. The work presented here was initially motivated by such a complex problem: SSMs in genetics, where *T*, which corresponds to DNA bases, can reach millions (see [13] and Section 6). This situation forces practitioners to collect fewer MCMC samples (which leads to increased variance) or use a simpler model and/or fewer data.

In addition to this computational burden, the issue of multi-modality in the posterior (i.e. the existence of many areas of high probability) is entirely unaddressed in pMCMC literature. Nevertheless, multi-modality can appear in genetics applications (see Section 6 and [13]). In such cases, pMCMC explores the posterior density slowly, a common problem for many MCMC methods which is known as slow mixing.

In order to handle the above limitations, this work follows two main directions. First, it proposes a new algorithm, which is able to sample from multi-modal distributions more efficiently than pMCMC. Second, it leverages the power of parallel hardware acceleration. More specifically, it uses a class of hardware devices called Field Programmable Gate Arrays (FPGAs), which offer massive parallel resources and have the distinctive characteristic that their hardware architecture can be fully customized according to the requirements of the implemented algorithm. Even more crucially, this work combines the above two approaches to further increase performance; algorithmic and hardware design are done jointly so that information about the nature of the underlying hardware can be exploited when designing the algorithm and vice versa. The contributions of this work are summarized below:

1. A new MCMC algorithm, denoted Population-based Particle MCMC – ppMCMC, which is an extension of pMCMC and which uses a population of chains instead of the single chain used by pMCMC. ppMCMC improves mixing for densities which are both analytically intractable and multi-modal (Section 4). A justification of why ppMCMC works, i.e. why it converges to the desired posterior distribution, is also provided.
2. A novel parallel hardware architecture for pMCMC, implemented on an FPGA, which exploits the parallelism inside the PF to improve sampling throughput (Section 5.2).
3. A novel parallel hardware architecture for ppMCMC, also implemented on an FPGA, which pipelines the computations of the multiple ppMCMC chains to increase the utilization of the PF datapath inside the FPGA (Section 5.3).

The new algorithm and the two FPGA samplers are applied to a large-scale inference problem in genetics – an SSM model of DNA methylation with unknown parameters (Section 6). This model can lead to uni-modal or multi-modal posteriors. In the former case, the FPGA pMCMC sampler is compared to optimized, state-of-the-art CPU and GPU implementations and it is found to be 6.4x–14.9x and 3.8x–30.8x faster respectively. In the latter case, the trade-off between the number of chains and the number of PF particles in ppMCMC is explored to maximize performance. When comparing sequential software implementations of the algorithms, ppMCMC is 1.96x faster than pMCMC. When comparing the FPGA implementations, the FPGA ppMCMC is up to 3.24x faster than the FPGA pMCMC sampler and up to 34.9x and 41.8x faster than the CPU pMCMC and GPU pMCMC samplers respectively.

The paper is organized as follows. Section 2 provides the necessary background on Bayesian inference, SSMs and pMCMC, as well as a short introduction on FPGAs, while Section 3 gives an overview of previous literature. Section 4 introduces the new ppMCMC algorithm and Section 5 describes the proposed FPGA architectures. Section 6 contains a description of the case studies used to evaluate the method, followed by Section 7 which presents the results of the evaluation. Finally, Section 8 concludes the paper.

## 2 Background

### 2.1 Bayesian inference

Bayesian inference is a simple framework, which consists in inferring the probability distribution of the unknown quantities of a probabilistic model given the known data. This is done by combining: 1) Information about the unknowns before any data are available, i.e. the prior distribution of the unknowns, 2) Information about the unknowns provided by the data, i.e. the likelihood of the unknowns. The inferred distribution is called the posterior distribution of the unknowns given the data. Practitioners are typically interested in evaluating or estimating expectations (integrals) over this posterior. The form of these integrals depends on the application and is outside the scope of this paper (for more information see [2] and [1]).

### 2.2 State-space models with unknown parameters

SSMs with unknown parameters are a class of Bayesian models, where a hidden sequence of states which evolves over time emits a sequence of observations, while the functions that define the relationships between states and observations have unknown parameters. More formally, let $X_{t}\in R^{n_{X}}$ be a random variable representing the state of the SSM at time step t∈{1,...,T}, where $n_{X}\in N$. Let $Y_{k}\in R^{n_{Y}}$ be another random variable representing the observation vector at time step *t*, where $n_{Y}\in N$. Time steps here can represent time, space, etc., depending on the application. An SSM is defined by the following set of equations:

(1) $$\begin{array}{c} X_{1}∼e(X_{1}) \end{array}$$

(2) $$\begin{array}{c} X_{t}∼f(X_{t}|X_{t-1},\theta ),\;\;t>1 \end{array}$$

(3) $$\begin{array}{c} Y_{t}∼g(Y_{t}|X_{t},\theta ),\;\;t>0 \end{array}$$

**Fig. 1:**
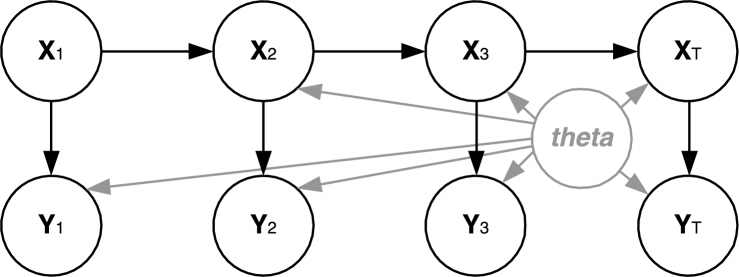
Hidden states, observations and unknown parameters of SSM.

Here, $e(X_{1})$ is the initial probability density, $f(X_{t}|X_{t-1},\theta )$ is the probability density of moving to the current state given the previous state (**transition density**) and $g(Y_{t}|X_{t},\theta )$ is the probability density of observing the current observation given the current state (**observation density**). The two densities also depend on a set of unknown parameters, $\theta \in R^{n_{\theta }}$ with $n_{\theta }\in N$. Usually, different parts of the ***θ*** vector are used in (2) and (3) but here the whole vector appears in both densities for simplicity. Symbol “∼” means distributed according to. Fig. 1 depicts the structure of an SSM.

From the standpoint of Bayesian inference, the unknown quantities that need to be inferred are 1) the hidden state sequence $X_{1:T}$ and 2) the unknown parameters ***θ***. It is assumed that the random variable representing the observation vector is known, i.e. $Y_{1:T}=y_{1:T}$, where $y_{1:T}$ is a known vector (also called the data). Therefore the goal of SSM inference is to infer the following joint posterior:

(4) $$\begin{array}{c} p(X_{1:T},\theta |Y_{1:T}=y_{1:T})=p(X_{1:T},\theta |y_{1:T})\propto \rho (\theta )\;\pi (X_{1:T}|\theta )\;\lambda (y_{1:T}|X_{1:T},\theta )\\ =\rho (\theta )\;e(X_{1})\;(\prod_{t=2}^{T}f(X_{t}|X_{t-1},\theta ))\;(\prod_{t=1}^{T}g(y_{t}|X_{t},\theta )) \end{array}$$

Here, ρ(θ) is the Bayesian prior density of the parameter ***θ***, $\pi (X_{1:T}|\theta )$ is the Bayesian prior density of the states and $\lambda (y_{1:T}|X_{1:T},\theta )$ is the likelihood of the data (observations) given the unknowns. The right-most part of the second line of the equation is easily derived from the densities of Section 2.2 (see [7] for details).

### 2.3 pMCMC: joint state and parameter estimation in SSMs

pMCMC (Algorithm 1) solves the SSM inference problem stochastically, i.e. by drawing samples from the joint posterior of equation (4). In other words, pMCMC generates a sequence of *N* joint samples ${\left(X_{1:T},\theta \right)}_{1:N}$, which are distributed according to $p(X_{1:T},\theta |y_{1:T})$.

At each loop iteration in line 9, pMCMC performs three steps: 1) It proposes a candidate sample $\theta^{⁎}$ for the unknown parameter based on the previous sample ***θ***, using a simple probability density q(⋅|⋅) (line 10). 2) It calls a PF algorithm (which will be examined later) to achieve two things: First, to generate a set of *P* samples from $p(X_{1:T}|y_{1:T},\theta^{⁎})$. One of these samples is selected randomly in line 12 and serves as the proposed state sequence sample $X_{1:T}^{⁎}$. Second, to produce an unbiased estimate of the quantity $l(y_{1:T}|\theta^{⁎})$, which is the likelihood of the data, given the SSM parameters. The estimate is denoted by $\overset{˜}{l}(y_{1:T}|\theta^{⁎})$ and it is necessary for the following step of the algorithm. The PF run is the most computationally intensive part of pMCMC. 3) It accepts the joint candidate sample $\left(X_{1:T}^{⁎},\theta^{⁎}\right)$ with probability min(1,a) where the acceptance ratio *a* is:

(5) $$\begin{array}{c} a=\frac{\rho (\theta^{⁎})\;\overset{˜}{l}(y_{1:T}|\theta^{⁎})\;q(\theta |\theta^{⁎})}{\rho (\theta )\;\overset{˜}{l}(y_{1:T}|\theta )\;q(\theta^{⁎}|\theta )} \end{array}$$

Otherwise the previous sample is replicated (lines 13–20). The first iteration (lines 2–7) only performs step 2 to initialize the sampler. The inputs of pMCMC are the number of MCMC iterations (*N*), the initial parameter sample ($\theta^{init}$) and the number of particles of the PF (*P*). The algorithm returns the history of samples Sample[1:N] and posteriors Posterior[1:N] after all *N* iterations have been completed.

The critical contribution of [3] is the proof that, as long as the likelihood estimates in the numerator and denominator of the ratio (5) are unbiased, the algorithm converges to the “correct” distribution, i.e. the posterior $p(X_{1:T},\theta |y_{1:T})$. The likelihood estimates provided by the PF are unbiased, so pMCMC samples from the “correct” posterior. Moreover, this is true regardless of the variance of the PF estimate.

As mentioned above, running a PF is necessary at each iteration of pMCMC in order to generate a candidate sample of the SSM states and an unbiased estimate of the SSM likelihood. PFs are a powerful class of methods used for state estimation in SSMs. A Bootstrap PF (Algorithm 2) is used here, as it is the most popular PF variant.

The inputs of Algorithm 2 are the number of particles (*P*) and the candidate $\theta^{⁎}$ (generated in the previous step of the pMCMC algorithm). The PF uses the set of *P* particles to estimate each state in the sequence $X_{1:T}$. At every iteration (line 8), it propagates particles to the next SSM time step using: 1) Sampling (lines 13–15) where the transition density (2) is used to sample particles for the next state, 2) Weighting (lines 16–17) where the observation density (3) is used to calculate weights for all particles. The weight quantifies the quality of a particle as an estimate of the next state, 3) Resampling (lines 9–12), where a new particle set is generated, in which particles with large weights are replicated many times and particles with low weights are discarded (equivalent to multinomial sampling). Resampling is critical for the stability of the PF (see [7]).

After all *T* time steps have finished, the ensemble of all particle sets $X_{1:T}^{1:P}$ is available. Also, the algorithm uses the computed weights to produce an estimate of the likelihood (line 19). This estimate is unbiased [7]. The outputs of the PF are used inside pMCMC. Specifically, a random particle sequence is chosen from the ensemble $X_{1:T}^{1:P}$ (line 12 of Algorithm 1) and the likelihood estimate is used in the acceptance step, as described above.

The main tuning parameter of pMCMC is the number of particles (*P*). With larger *P*, each pMCMC iteration becomes more time-consuming but the variance of the likelihood estimate decreases. The latter leads to faster pMCMC mixing [14], i.e. faster exploration of the posterior, since pMCMC moves closer to an exact MCMC algorithm. Therefore, there is a tradeoff between the runtime of the PF and the mixing of pMCMC.

The choice of the proposal distribution q(⋅|⋅) is also a tuning parameter of the algorithm. In practice, a Gaussian proposal is often used and its variance is adjusted to maximize mixing. This is the approach followed in this article; a multivariate Gaussian proposal is used and its covariance matrix is tuned for each examined SSM posterior (the exact covariance matrices can be found in Section 6.1). Finally, like in all MCMC algorithms, it is usual to discard an initial chunk of the *N* samples as burn-in, in order to make sure the sampler has converged to the target distribution. This practice is followed in this article, as will be detailed in Section 6.1.

### 2.4 Field Programmable Gate Arrays

**Fig. 2:**
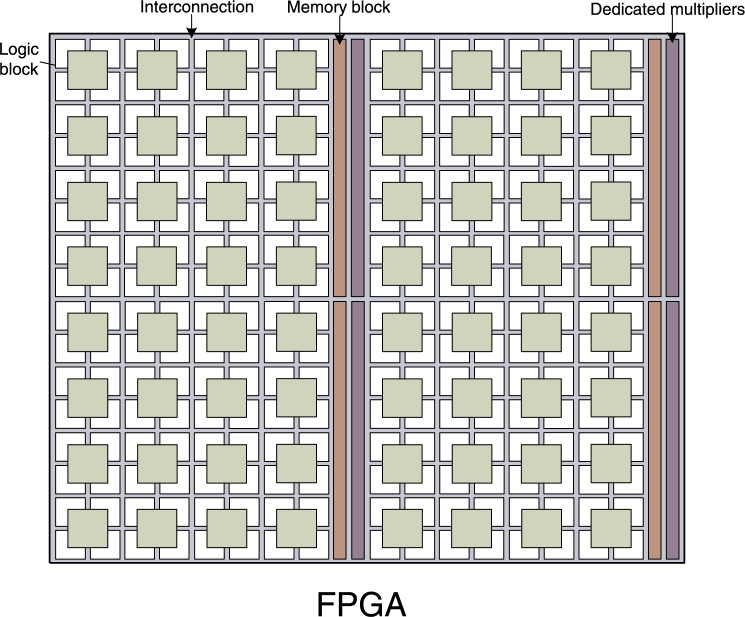
Simplified FPGA architecture.

FPGAs are a powerful and unique class of digital hardware devices, which are typically used as accelerators for computationally intensive algorithms. FPGAs fundamentally differ from other hardware devices such as Central Processing Units (CPUs) or Graphics Processing Units (GPUs). While CPUs and GPUs have fixed hardware architectures defined before the chip is manufactured, FPGAs consist of a re-programmable “fabric” upon which any custom hardware architecture can be mapped [15]. As Fig. 2 shows, the FPGA fabric consist of an array of programmable logic blocks and a hierarchy of interconnects which allow the blocks to be wired together. Each block can implement a simple boolean function. By connecting the blocks together any digital circuit can be implemented. Moreover, modern FPGAs are equipped with dedicated units to perform common arithmetic operations, as well as several on-chip memory blocks.

FPGAs allow the designer to tailor the hardware to the specific characteristics of the application. The number and granularity of parallel processing elements, the kinds of arithmetic operators embedded in these elements, the amount and architecture of cache memories and the arithmetic precision of computations are all customizable. Thus FPGAs can exploit non-obvious and/or limited parallelism, maximize pipelining efficiency and adapt the memory architecture to the access pattern of the algorithm. For example, while a GPU architecture has a pre-defined amount of on-chip memory per processing core, an FPGA can allocate a custom amount of on-chip memory to each “processing element” or even allow all elements to access all the memory, depending on the requirements of the targeted algorithm. Moreover, the programming of the fabric can be done as many times as desired and even during runtime and/or partially. All this flexibility can lead to significantly higher performance [15] and lower power consumption [16] compared to fixed-architecture devices (CPUs, GPUs). Nevertheless, FPGA design requires specialized programming skills and longer development times than CPU and GPU coding, despite the recent emergence of various high level tools [17]. In addition, CPUs and GPUs remain competitive for the types of problems they are designed for; sequential/conditional code and Single-Instruction-Multiple-Data (SIMD) algorithms respectively.

This is the main reason which has led hardware manufacturers to develop devices that combine traditional CPUs with a custom FPGA fabric on the same chip [18]. These devices combine the benefits of both worlds; they allow the sequential and non-critical part of the targeted algorithm (including libraries that might be hard to migrate to an FPGA) to be run on the CPU, while the custom fabric is responsible for the parallel, computationally intensive part. Different architectures can be loaded on the fabric depending on the algorithm. It is expected that these hybrid chips will play an increasingly important role in the data center and high performance computing markets, where the demand for high throughput and low power is strong. Algorithms like MCMC and PF are commonly used in these computing environments, since they are fundamental tools for numerous large-scale scientific applications.

## 3 Related work

The first work on parallel acceleration of pMCMC using GPUs was published in 2012 [19]. The sample and weight steps of the PF were parallelized and a systematic resampling algorithm was implemented using a parallel prefix-sum technique. No details on the GPU device and the speedups over software were presented and the case studies were small SSMs (T=100). Therefore the efficiency of the implementation cannot be evaluated. Moreover, the limited number of states and particles is not representative of real, large-scale problems where hardware acceleration is needed.

A more recent work [10] proposed an automated tool (LibBi) for SSM inference on CPUs and GPUs which supported pMCMC. LibBi provides a domain-specific language for SSMs and a parallel processing back-end (combining MPI, OpenMP, SSE and CUDA). Nevertheless, the language is inflexible in terms of the definable transition/observation densities; some popular densities and several common types of computations are not supported. Some of these disadvantages can be mitigated by the fact that the tool is openly available and can be modified. [10] also contains limited results on applying the framework to a Lorenz '96 SSM with T=40 and P=8192. The evaluation showed that using the framework on a CPU with OpenMP and SSE optimizations was 5x faster than running sequential C++ code; the combined CPU/GPU version (with CUDA) provided an extra 4x speedup.

The work presented here is the first to use FPGAs to accelerate pMCMC, taking advantage of the unique features of the platform (e.g. custom pipelining) to accelerate sampling. It is also the first to use a modified, parallelized version of the Residual Systematic Resampling algorithm inside the PF (first introduced in [20]). Finally, this work is the first to apply pMCMC to large SSMs, where *T* and *P* scale to many thousands.

In the algorithmic level, [21] and [14] have proposed ways to optimize *P* based on the tradeoff between PF runtime and pMCMC mixing, which was mentioned above. Depending on assumptions, they recommend that *P* should be chosen so that the variance of the likelihood estimate is between 1.0 and 3.2, in order to maximize pMCMC's mixing per second (the “exploration” of the state space that pMCMC achieves per unit of computing time). Nevertheless, the following issues remain unaddressed: 1) When optimizing the value of *P*, both [21] and [14] assume that the PF's runtime grows proportionately to *P*; this is not true when using parallel implementation of the PF algorithm. 2) The use of multiple MCMC chains to improve mixing has not been examined, although it is clear that pMCMC can mix very slowly for multi-modal posteriors, even with large *P*.

This work tackles the first issue by taking into account the performance of parallel PF implementations (either in the FPGA or in the CPU/GPU) when optimizing the value of *P*. In order to address the second issue, a novel pMCMC sampler (denoted ppMCMC) is introduced, along with an accompanying FPGA architecture. ppMCMC improves mixing for multi-modal posteriors by utilizing multiple MCMC chains instead of the one used in pMCMC. The “correctness” of the new algorithm, i.e. the fact that it samples from the correct SSM posterior, is also justified. Moreover, in contrast to [21] and [14], where *P* is the only optimized parameter, the present work jointly optimizes *P* and *M* (the number of MCMC chains in ppMCMC) in order to find the combination that maximized mixing per second.

Finally, this is the first use of pMCMC and FPGAs for methylation analysis (see the case study of Section 6). So far, pMCMC was considered intractable for such problems and approximate methods were used [22], leading to bias in the resulting estimates.

## 4 ppMCMC: a new MCMC algorithm

### 4.1 Summary

This section presents a novel MCMC algorithm, which extends pMCMC by adding auxiliary MCMC chains in order to improve mixing in scenarios where the SSM posterior (4) is multi-modal, such as the one presented in Section 6. Multi-modality is the existence of multiple areas of high probability in the posterior [23]. Standard pMCMC faces the well-known issue of slow mixing when sampling from such posteriors. This issue is common in many single-chain MCMC methods. The sampler tends to “get stuck” in one of the modes of the posterior and rarely manages to move to another mode [23].

The new algorithm is called Population-based Particle MCMC (ppMCMC) and it is a combination of two existing MCMC methods (Population-based MCMC [23] and pMCMC). The pseudo-code of ppMCMC is given in Algorithm 3. It makes use of a population of *M* pMCMC chains. At each iteration (loop in line 10), ppMCMC performs two kinds of operations for all chains.

The first is the *update operation* (loop in line 12), during which the next MCMC sample of each chain is generated, using the same three steps described for pMCMC: 1) Propose a candidate unknown parameter sample $\theta^{⁎}$ (line 13) using density $q_{j}(\cdot |\cdot )$ for chain j∈{1,...,M}. Like in pMCMC, multivariate Gaussian proposal distributions are used in ppMCMC. The covariance matrix of these distributions can be tuned. 2) Run a PF to propose a candidate state sequence sample $X_{1:T}^{⁎}$ and an estimate of the likelihood (lines 14–15). 3) Accept the joint candidate with probability $min(1,a_{j})$ for chain j∈{1,...,M}. Otherwise repeat the previous sample (lines 16–24). The difference compared to pMCMC is the use of a different acceptance ratio for each chain (step 3). This leads each chain to sample from a different distribution. Chain 1 samples from the “correct” posterior (4). The other chains sample from “smoothed” versions of (4) and their mixing is faster than the mixing of the first chain, as will be explained in Section 4.2. Only the samples of the first chain are kept.

The second operation within each iteration of ppMCMC is the *exchange operation* (loop in line 26), which was not used in pMCMC. During this operation, pairs of neighboring chains (e.g. (1,2), (3,4), etc.) exchange their MCMC samples with probability $min(1,e_{q})$ (for pair (q,q+1),q∈{1,...,M−1}). Sample exchanges help the algorithm mix faster because they push samples from the auxiliary chains to the first chain, helping it to escape from local modes. This is explained in more detail below.

### 4.2 Update and exchange operations

#### 4.2.1 Updates

During the update operation for chain j∈{1,...,M}, the following acceptance ratio is used:

(6) $$\begin{array}{c} a_{j}=\frac{\rho (\theta^{⁎})\;\overset{˜}{l}{\left(y_{1:T}|\theta^{⁎}\right)}^{\frac{1}{Temp_{j}}}\;q_{j}(\theta_{j}|\theta^{⁎})}{\rho (\theta_{j})\;\overset{˜}{l}{\left(y_{1:T}|\theta_{j}\right)}^{\frac{1}{Temp_{j}}}\;q_{j}(\theta^{⁎}|\theta_{j})},\;\;j\in \{1,...,M\} \end{array}$$

where $Temp_{j}$ is the temperature of chain *j* and $1=Temp_{1}<Temp_{2}<...<Temp_{M}<\infty$. Therefore, only chain j=1 uses the acceptance ratio of pMCMC (given in (5)) and thus samples from the “correct” SSM posterior. The remaining (auxiliary) chains (j∈{2,...M}) use a tempered acceptance ratio, i.e. the estimated likelihood in the numerator and the denominator is raised to the power of $\frac{1}{Temp_{j}}$ for chain *j*. This leads the auxiliary chains to sample from some set of “smoothed” (closer to uniform) versions of the “correct” SSM posterior. Chains with smoother densities mix faster, since it is easier for the sampler to jump between modes when the modes are smoothed.

Although applying a temperature to the likelihood is a well-known technique in population-based methods, in the case of ppMCMC it is not clear what the target distribution of each tempered chain is. The term $p(\theta )\overset{˜}{p}{\left(y_{1:T}|\theta \right)}^{\frac{1}{Temp_{j}}}p(X_{1:T}|y_{1:T},\theta )$ (which contains the likelihood estimate) is used in the acceptance ratio of chain *j* but this does not lead the chain to converge to the posterior $p(\theta )p{\left(y_{1:T}|\theta \right)}^{\frac{1}{Temp_{j}}}p(X_{1:T}|y_{1:T},\theta )$, as one would intuitively expect after comparing to the pMCMC acceptance ratio in (5).

According to the theory presented in [6], a pMCMC chain converges to a target distribution provided that unbiased estimates of the distribution's density are used in the numerator and denominator of the acceptance ratio. Nevertheless, the term $p(\theta )\overset{˜}{p}{\left(y_{1:T}|\theta \right)}^{\frac{1}{Temp_{j}}}p(X_{1:T}|y_{1:T},\theta )$ is not an unbiased estimator of $p(\theta )p{\left(y_{1:T}|\theta \right)}^{\frac{1}{Temp_{j}}}p(X_{1:T}|y_{1:T},\theta )$: Running a PF on the given SSM produces an unbiased estimate $\overset{˜}{p}(y_{1:T}|\theta )$ of the likelihood (i.e. $E[\overset{˜}{p}(y_{1:T}|\theta )]=p(y_{1:T}|\theta )$). However, applying the temperature after the likelihood estimate is generated (i.e. finding $\overset{˜}{p}{\left(y_{1:T}|\theta \right)}^{\frac{1}{Temp_{j}}}$) does not maintain unbiasedness with respect to the “correct” tempered likelihood (i.e. $p{\left(y_{1:T}|\theta \right)}^{\frac{1}{Temp_{j}}}$). In more detail, because the function $x\mapsto x^{\frac{1}{Temp_{j}}}$ is concave for $Temp_{j}\geq 1$, applying Jensen's inequality [24] leads to the following:

(7) $$\begin{array}{c} E[\overset{˜}{p}{\left(y_{1:T}|\theta \right)}^{\frac{1}{Temp_{j}}}]\leq E{\left[\overset{˜}{p}(y_{1:T}|\theta )\right]}^{\frac{1}{Temp_{j}}}=p{\left(y_{1:T}|\theta \right)}^{\frac{1}{Temp_{j}}} \end{array}$$

This is true for all ppMCMC chains since all temperatures are greater or equal to one. Equality holds only for $Temp_{j}=1$. Therefore, unbiased estimates of the “correct” tempered likelihood densities $p{\left(y_{1:T}|\theta \right)}^{\frac{1}{Temp_{j}}}$ (and thus the respective posterior densities) can be acquired only when $Temp_{j}=1$ (i.e. only in the case of the first chain). Nevertheless, this does not mean that the tempered chains do not converge to *any* target distribution. In fact, chain *j* converges to the distribution whose density is unbiasedly estimated by $p(\theta )\overset{˜}{p}{\left(y_{1:T}|\theta \right)}^{\frac{1}{Temp_{j}}}p(X_{1:T}|y_{1:T},\theta )$. The densities of these distribution can be written as:

(8) $$\begin{array}{c} p_{j}(X_{1:T},\theta |y_{1:T})=p(\theta )\;E[\overset{˜}{p}{\left(y_{1:T}|\theta \right)}^{\frac{1}{Temp_{j}}}]\;p(X_{1:T}|y_{1:T},\theta ),\;\;\;j\in \{1,...,M\} \end{array}$$

These are the actual target densities of the *M* chains of the ppMCMC algorithm. Only for the first chain the density is equal to the “correct” tempered posterior (with $Temp_{1}=1$) and also to the “correct” SSM posterior, i.e. $p_{j}(X_{1:T},\theta |y_{1:T})=p(X_{1:T},\theta |y_{1:T})$. The key point here is that it is not necessary for the auxiliary chains to sample from the set of “correct” tempered posteriors, since their samples are not kept. Only the samples of the first chain are kept because they are the ones distributed according to the desired, “correct” SSM posterior. The auxiliary chains are only employed to help the first chain mix faster; they need to explore the distribution space quickly (and therefore their target distributions need to be closer to uniform) and occasionally feed the first chain with samples through exchange moves. These samples help the first chain escape from local modes, as will be explain shortly. It is therefore enough for the auxiliary chains to sample from *some* set of tempered versions of the SSM posterior (and not necessarily from the “correct” set of tempered posteriors). The densities in equation (8) provide this tempering effect and therefore fulfill their purpose, i.e. they move fast in the distribution space and help the first chain mix faster through exchange moves.

In fact, the term “correct” is only used here for reasons of clarity; there is no reason to believe that the “correct” densities $p{\left(y_{1:T}|\theta \right)}^{\frac{1}{Temp_{j}}}$ are the best candidates for use in auxiliary chains (with respect to the mixing gains they offer). On the other hand, this does not mean that *any* density would serve as a good auxiliary density, e.g. uniform auxiliary densities would not help the mixing of the first chain because they are not concentrated around the true modes. In other words, *some* smoothing must be applied to the true densities but the form of the *optimal* auxiliary densities is not known.

Regarding the choice of the temperature values, they often follow a geometric or additive progression in MCMC literature, i.e. $\frac{T_{i+1}}{T_{i}=constant}$ or $T_{i+1}-T_{i}=constant$, although other choices have also been considered [25]. In the evaluation section of this article, ppMCMC temperatures follow an additive progression. The exact temperature values are given in Section 6.

#### 4.2.2 Exchanges

In order to exploit the tempering process described above to improve mixing, ppMCMC makes use of sample *exchanges*, which attempt to swap samples between chains at each iteration. Exchange moves are attempted between chain pairs (1,2),(3,4),… or chain pairs (2,3),(4,5),… (neighboring chains) in a rotating order. As mentioned above, the exchange moves push MCMC samples from the high-temperature chains, which are closer to the uniform distribution, to the lower-temperature chains, which are closer to the “correct” target distribution. Eventually samples reach the first chain which samples from the “correct distribution” and help it escape from local modes. The exchange acceptance ratio between chains (q,r) is:

(9) $$\begin{array}{c} e_{q}=\frac{\overset{˜}{l}{\left(y_{1:T}|\theta_{r}\right)}^{\frac{1}{Temp_{q}}}\;\overset{˜}{l}{\left(y_{1:T}|\theta_{q}\right)}^{\frac{1}{Temp_{r}}}}{\overset{˜}{l}{\left(y_{1:T}|\theta_{q}\right)}^{\frac{1}{Temp_{q}}}\;\overset{˜}{l}{\left(y_{1:T}|\theta_{r}\right)}^{\frac{1}{Temp_{r}}}} \end{array}$$

where q∈{1,...,M−1}, r=q+1 and $\left(X_{1:T}^{q},\theta^{q}\right)$ and $\left(X_{1:T}^{r},\theta^{r}\right)$ are the current samples of chains *q* and *r* respectively. It has to be noted that the above ratio requires no additional PF runs (all the values are already known from the preceding update steps).

It is important to justify why the above exchange move fulfills the requirements of the theory of pMCMC [6] with regards to maintaining the correct target distributions of the two chains. The exchange step is equivalent to a Metropolis update where the updated state is the joint state of both chains (with indexes *q* and *r*). According to [6], a Metropolis update maintains the target distribution as long as the numerator and denominator of the acceptance ratio are unbiased estimates of the target density. In the case of the exchange step (and focusing only on the numerator for simplicity), this means that the product $p(\theta^{r})\;\overset{˜}{p}{\left(y_{1:T}|\theta^{r}\right)}^{\frac{1}{Temp_{q}}}\;p(X_{1:T}^{r}|y_{1:T},\theta^{r})\;p(\theta^{q})\;\overset{˜}{p}{\left(y_{1:T}|\theta^{q}\right)}^{\frac{1}{Temp_{r}}}\;p(X_{1:T}^{q}|y_{1:T},\theta^{q})$ (denoted by *U*) has to be an unbiased estimate of the product of the target densities of the two chains (which were given in equation (8)). It is easy to show that this is the case. Since $p(\theta^{r})$, $p(X_{1:T}^{r}|y_{1:T},\theta^{r})$, $p(\theta^{q})$ and $p(X_{1:T}^{q}|y_{1:T},\theta^{q})$ are all zero-variance estimators, the following is true:

(10) $$\begin{array}{c} E[U]=E[p(\theta^{r})\;\overset{˜}{p}{\left(y_{1:T}|\theta^{r}\right)}^{\frac{1}{Temp_{q}}}\;p(X_{1:T}^{r}|y_{1:T},\theta^{r})\;p(\theta^{q})\\ \overset{˜}{p}{\left(y_{1:T}|\theta^{q}\right)}^{\frac{1}{Temp_{r}}}\;p(X_{1:T}^{q}|y_{1:T},\theta^{q})]=p(\theta^{r})\;p(X_{1:T}^{r}|y_{1:T},\theta^{r})\;p(\theta^{q})\\ p(X_{1:T}^{q}|y_{1:T},\theta^{q})\;E[\overset{˜}{p}{\left(y_{1:T}|\theta^{r}\right)}^{\frac{1}{Temp_{q}}}\;\overset{˜}{p}{\left(y_{1:T}|\theta^{q}\right)}^{\frac{1}{Temp_{r}}}] \end{array}$$

Moreover, it is known that $\overset{˜}{p}{\left(y_{1:T}|\theta^{r}\right)}^{\frac{1}{Temp_{q}}}$ and $\overset{˜}{p}{\left(y_{1:T}|\theta^{q}\right)}^{\frac{1}{Temp_{r}}}$ are independent estimators (since they are generated by two independent PFs, each assigned to its own MCMC chain). Therefore, their product is equal to the product of their expectations. Combining this with equation (10), it is clear that:

(11) $$\begin{array}{c} E[U]=p(\theta^{r})\;p(X_{1:T}^{r}|y_{1:T},\theta^{r})\;p(\theta^{q})\;p(X_{1:T}^{q}|y_{1:T},\theta^{q})\\ E[\overset{˜}{p}{\left(y_{1:T}|\theta^{r}\right)}^{\frac{1}{Temp_{q}}}]\;E[\overset{˜}{p}{\left(y_{1:T}|\theta^{q}\right)}^{\frac{1}{Temp_{r}}}]=p_{q}(X_{1:T},\theta^{r}|y_{1:T})\;p_{r}(X_{1:T},\theta^{q}|y_{1:T}) \end{array}$$

The last line of the equation is true due to equation (8) and proves that the product *U* is an unbiased estimate of the of the product of the target densities of the two chains.

## 5 FPGA architectures for pMCMC and ppMCMC

This section presents novel FPGA architectures for pMCMC and ppMCMC, which exploit the inherent parallelism of each algorithm.

### 5.1 Parallelism in the algorithms

In pMCMC, the available parallelism is O(P), since all particles inside the PF can be processed in parallel (although resampling requires communication between parallel processes). The *N* iterations of pMCMC are strictly sequential. In ppMCMC, the available parallelism increases to O(M⋅P) due to the existence of *M* MCMC chains which can be updated independently (although exchanges require inter-chain communication). The independence of the update operations can be exploited to pipeline computations in the PF datapath.

### 5.2 pMCMC architecture

**Fig. 3:**
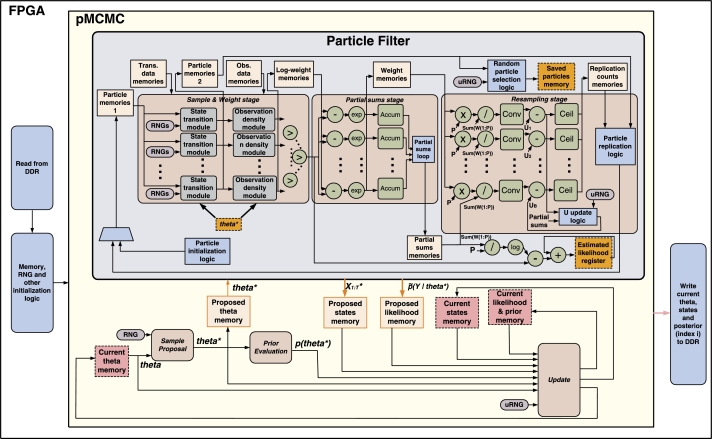
FPGA architecture for pMCMC. The orange memory blocks are inputs/outputs of the PF. The pink memory blocks are outputs of pMCMC. (For interpretation of the references to color in this figure legend, the reader is referred to the web version of this article.)

The pMCMC architecture is illustrated in Fig. 3. The pMCMC block within the architecture comprises all the necessary parts of an MCMC sampler plus a PF block. The PF block comprises three stages: Sample & Weight, Partial sums and Resampling (grouped in a different way compared to the description of Section 2.3 for implementation reasons). The output MCMC samples are stored in external (off-chip) memory, which is not shown in the figure (more details below).

At every pMCMC iteration (i∈{1,...,N}), the current (latest) ***θ*** sample is read from the *Current theta memory* and sent to the *Sample Proposal block*, which samples from $q(\theta^{⁎}|\theta )$. The candidate $\theta^{⁎}$ is written to the *Proposed theta memory* and forwarded to the PF. The latter returns the estimated likelihood $\overset{˜}{l}(y_{1:T}|\theta^{⁎})$ and a randomly selected state sample $X_{1:T}^{⁎}=X_{1:T}^{p}$; these are written to the respective memories. The *Prior Evaluation block* computes the prior $\rho (\theta^{⁎})$. The *Update block* accepts or rejects $\left(X_{1:T}^{⁎},\theta^{⁎}\right)$ based on (5), using several input values. If the update is successful, the candidate sample and the proposed likelihood and prior values are written to the respective current memories (equivalent to Sample[i] and Posterior[i] in Algorithm 1). Otherwise, the current memories remain unchanged (keeping Sample[i−1] and Posterior[i−1]). After each iteration, the contents of the current memories are sent to the off-chip memory. The above steps are repeated for all pMCMC loop iterations.

*PF block:* This block is activated once per pMCMC iteration. The sample, weight and resampling operations are repeated *T* times, once for every time step, with the order of operations changed compared to Algorithm 2. Each step has to process all *P* particles (loops in lines 9, 13 and 16). Computations for each particle are independent, therefore they are parallelized and pipelined. The architecture's parallelism (number of parallel modules) is the same for all steps and is denoted by *B*. Each parallel module processes $\frac{P}{B}$ particles. Each memory that feeds the modules is partitioned into *B* sub-memories and each sub-memory is assigned to one module.

The transition and weight operations are implemented inside the *Sample & Weight* stage of the architecture. At each step *t*, particles $X_{t-1}^{1:P}$ are read from the *Particle memories* and fed to the *B State transition block*. These sample from (2) using $\theta^{⁎}$ as the parameter. The output is written to *Particle memories 2* and passed to the *B* parallel observation density modules to compute the log-weights $log(W_{t}^{1:P})$ using (3) (working with log-weights helps avoid numerical issues). Again, the candidate $\theta^{⁎}$ is used as the parameter in (3). The log-weights are written to the *Log-weight memories* and passed to a comparator tree to find the maximum log-weight.

The following two stages of the architecture implement resampling (equivalent to lines 9–12 of Algorithm 2), using the Residual Systematic Resampling (RSR) algorithm [20]. RSR requires the partial sums of weights for each parallel processing module, i.e. module l∈{1,...,B} requires the sum $\sum_{k=1}^{(l-1)\frac{P}{B}}W_{t}^{k}$. RSR outputs the replication counts of all particles ($r^{1:P}$), i.e. the number of times each particle is replicated.

In the *Partial Sums stage*: 1) The maximum log-weight is used for renormalization to avoid numerical issues, 2) Log-weights are exponentiated to find the weights and 3) *B* parallel accumulators produce the sums of weights of each module $\sum_{k=(l-1)\frac{P}{B}+1}^{l\frac{P}{B}}W_{t}^{k},\;l\in \{1,...B\}$. These are then used to generate the partial sums and the sum of all weights. In the *Resampling stage*, the RSR architecture proposed in [20] is used. *B* pipelined datapaths compute the replication counts and write them to the respective memory. Details can be found in [20]. After all counts are stored, they are read sequentially and particle *k* is copied from *Particle memories 2* to *Particle memories 1* $r^{k}$ times. The use of two memories is necessary to avoid overwriting particles before their replication counts are examined. The process cannot be parallelized by replicating each bunch of $\frac{P}{B}$ particles independently, since the total number of replications of a bunch might exceed the memory partition assigned to it ($\frac{1}{B}$th of the total memory). For instance, if P=10 and B=2, each parallel block would process 5 replication counts/particles and have a particle memory of length 5 to its disposal. If particle k=1 has a replication count of 6, this means that the first parallel block does not have enough memory space to replicate the particle and needs to access the second memory block. The replication process was not examined in [20].

In parallel to resampling, the mean of the current weights is computed (using the partial sums), its logarithm is found, the renormalization (mentioned above) is inverted and the result is fed to an accumulator (below the *Resampling stage* in Fig. 3). After the PF stages have been repeated *T* times, this accumulator outputs the final estimate of the log-likelihood: $log(\overset{˜}{l}(y_{1:T}|\theta^{⁎})=\sum_{t=1}^{T}log(\frac{1}{P}\sum_{k=1}^{P}W_{t}^{k})$. This is written to the *Proposed likelihood memory*. Also, after *T* time steps the *Saved particles memory* contains a randomly selected set of particles, i.e. the proposed state $X_{1:T}^{⁎}$, which is copied to the *Proposed states memory*.

All memories have constant sizes, based on user-defined parameters (maximum states $T_{max}$, maximum particles $P_{max}$, $n_{\theta }$, $n_{X}$ and $n_{y}$).

### 5.3 ppMCMC architecture

The ppMCMC architecture is based on the pMCMC architecture but differs in two ways: 1) It permits many chains (*M*) to be processed concurrently by the PF datapath, 2) It performs exchange moves between chains. The *Proposed theta*, *Proposed states*, *Proposed likelihood*, *Current theta*, *Current states*, *Current likelihood* and *Prior memories* are replicated $M_{max}$ times (maximum number of chains, set by the user). This way, each chain has its separate memory and can be updated and exchanged without interfering with the other chains.

**Fig. 4:**
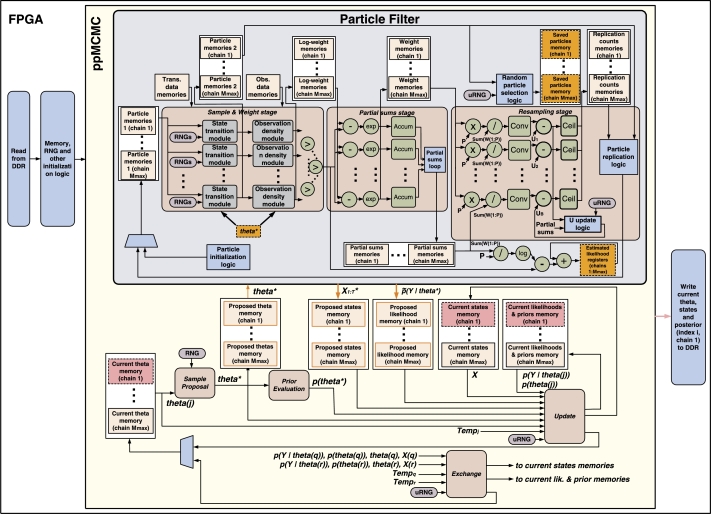
FPGA architecture for ppMCMC.

The ppMCMC architecture is shown in Fig. 4. The *Sample proposal block* proposes $\theta^{⁎}$ samples for all *M* chains and writes them to the respective memories. When it finishes, the PF computes the likelihoods and state samples for all chains using coarse-grain pipelining (see next paragraph). Only one PF block is instantiated (as in pMCMC); all chains are processed by this one block. This is followed by the *Update* and *Exchange blocks* which use equations (6) and (9) to perform the respective operations. The *Update block* needs an extra input compared to pMCMC – the chain temperature $Temp_{j}$. The *Exchange block* attempts swaps between pairs of chains as soon as both chains have been updated by the *Update block* (i.e. it does not wait for all chain to be updated as was the case in Algorithm 3).

*Coarse-grain pipelining in the PF:* The PF block is the same as in pMCMC (i.e. a single block) but exploits the independence of ppMCMC chains to increase datapath utilization. Running the PF for a single chain requires traversing the datapath *T* times, with each traversal comprising the *Sample & Weight*, *Partial sums* and *Resampling* stages. Each traversal has to wait until the previous one finishes; also, each stage can start only after the previous stage finishes. This means that, with one chain (as in the case of pMCMC), only one of the stages is utilized at a given moment. Let $Lat_{sw}$, $Lat_{ps}$ and $Lat_{re}$ be the latencies of the three stages for one time step (for all *P* particles). Then the total datapath latency is $Lat_{sw}+Lat_{ps}+Lat_{re}$. Without pipelining, the total latency for *M* chains (with *T* time steps each) is $M\cdot T\cdot (Lat_{sw}+Lat_{ps}+Lat_{re})$.

**Fig. 5:**
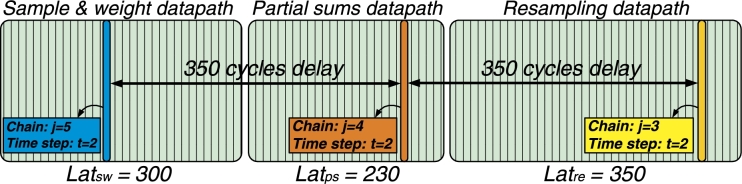
Coarse-grain pipelining in ppMCMC architecture: Resampling has the largest latency, thus a new chain is fed to the PF datapath every *Lat*_*re*_ = 350 cycles. Chains *j* = 3:5 are in the datapath, all of them at time step *t* = 2. Chain *j* = 4 will start Resampling one cycle after chain *j* = 3 finishes Resampling.

The ppMCMC architecture coarsely pipelines chain computations, exploiting the fact that the PF run of chain *j* does not need to wait for the PF run of chain j−1 to finish. Multiple tasks are processed in the PF at the same time, with the following order: First, time step t=1 of chain j=1 is processed, followed by time step t=1 of chain j=2, etc. When all the first time steps have finished, the second time steps (t=2) are fed to the PF for all chains and so on. By this change in the order of operations, the available parallelism is exposed. Pipelining is coarse-grain, since no PF stage is shared by two chains at the same time (to avoid significant control overheads); each chain waits for the previous one to finish a stage before entering the same stage. Using this technique (Fig. 5), a new chain can be fed to the datapath every $max(Lat_{sw},Lat_{ps},Lat_{re})$ cycles, bringing the total latency for *M* chains (*T* time steps each) to $M\cdot T\cdot max(Lat_{sw},Lat_{ps},Lat_{re}))$.

*Resource overheads:* In order to run many “virtual” PFs on the single PF hardware block simultaneously, multiple memories are used for all PF-accessed variables. The ppMCMC architecture uses $M_{max}$ *Particle*, *Log-weight*, *Weight*, *Saved particles*, *Replication counts* and *Partial sums* memories (each partitioned into *B* blocks as in pMCMC). The *Exchange block* is an additional overhead but takes few resources (see Section 7).

### 5.4 Performance models

The latency of the *Sample & weight stage* in pMCMC is:

(12) $$\begin{array}{c} Lat_{sw}=C_{1}+ceil(log(B))\cdot Lat_{comp}+\lceil \frac{P}{B}\rceil \end{array}$$

where $C_{1}$ is the (application dependent) latency of the parallel modules, the second term is due to the comparator tree and $\left\lceil \frac{P}{B}\right\rceil$ is the latency for passing *P* particles through the parallel modules. The latency of the *Partial sums stage* is:

(13) $$\begin{array}{c} Lat_{ps}=C_{2}+\lceil \frac{P}{B}\rceil \cdot Lat_{add}+B\cdot Lat_{add} \end{array}$$

where $C_{2}$ is the latency of the parallel modules, $\lceil \frac{P}{B}\rceil \cdot Lat_{add}$ is the latency for passing *P* particles through the datapath ($Lat_{add}$ is the adder latency) and $B\cdot Lat_{add}$ is the latency of the partial sums loop. The latency of the *Resampling stage* is:

(14) $$\begin{array}{c} Lat_{re}=C_{3}+\lceil \frac{P}{B}\rceil +P\cdot Lat_{rep} \end{array}$$

where $C_{3}$ is the latency of the parallel modules, $\left\lceil \frac{P}{B}\right\rceil$ is the latency for passing *P* particles through the datapath and $P\cdot Lat_{rep}$ is the particle replication latency ($Lat_{rep}$ is the mean replication count). One PF run in pMCMC costs:

(15) $$\begin{array}{c} Lat_{pf\_pMCMC}=\lceil \frac{P}{B}\rceil +T\cdot (Lat_{sw}+Lat_{ps}+Lat_{re}) \end{array}$$

where $\left\lceil \frac{P}{B}\right\rceil$ is due to particle initialization.

In ppMCMC, processing one time step for all chains costs:

(16) $$\begin{array}{c} Lat_{ts\_ppMCMC}=C_{4}+M\cdot max(Lat_{sw},Lat_{ps},Lat_{re})+(Lat_{sw}+Lat_{ps}+Lat_{re}-max(Lat_{sw},Lat_{ps},Lat_{re})) \end{array}$$

where $C_{4}$ is due to minor computations before each time step. The latency of processing all *T* time steps of all *M* chains is:

(17) $$\begin{array}{c} Lat_{pf\_ppmcmc}=M\cdot \lceil \frac{P}{B}\rceil +T\cdot Lat_{ts\_ppmcmc} \end{array}$$

where $M\cdot \lceil \frac{P}{B}\rceil$ cycles are needed for particle initialization.

## 6 Case study: SSMs for DNA methylation profiling

In order to evaluate the proposed algorithm and architectures of the previous sections, a large-scale synthetic SSM case study which focuses on DNA methylation is used. This case study is designed to demonstrate the benefits of the algorithmic and hardware architecture novelties proposed in this article, using a realistic, demanding application.

Methylation is a biochemical process which happens in specific positions of the DNA and is associated with a number of diseases. The positions where methylation happens cannot be detected directly. Methylation analysis consists in discovering these “hidden” positions by applying a process called sodium bisulfate treatment to the DNA bases, several times. The treatment is essentially a test for methylation, whose output is either a successful or a failed detection. Nevertheless, the test can be largely unreliable and noisy in many cases and further analysis is needed to successfully detect methylation. Methylation analysis can be applied to single-tissue of multi-tissue DNA. The latter case occurs when processing mixed tissue, e.g. whole-blood samples.

Methylation data can be simulated using SSM models; the hidden methylated states can be represented by the hidden states of the SSM and SSM inference can be used to estimate them based on known observations (i.e. the bisulfate treatment test results). SSMs have the advantage that they can represent dependencies between neighboring states using the transition density; this is important for methylation modeling, since the methylation state of a DNA base depends on the state of the neighboring bases.

Here, two synthetic DNA methylation data sets are simulated using SSMs: 1) A single-tissue data set, 2) A multi-tissue data set. First, the real methylation problem setting will be presented, followed by a description of how this is mapped to an SSM. In each case, the DNA sequence is assumed to have a length of *T* bases, where base t∈{1,...,T} has probability $p_{t}$ of being methylated. The goal is to estimate these probabilities using the results of methylation tests on 4 biological replicates k∈{1,...,4} (i.e. individuals with identical DNA sequences). At base *t* and for replicate *k*, a sodium bisulfate treatment test is applied $n_{kt}$ times. Out of these tests, $y_{kt}$ are successful. The physical position of DNA base *t* is $\delta_{t}$. The physical position is needed because typically some gaps exist between bases in real data sets. The goal of the analysis is to discover the probability $p_{t}$ that position *t* is methylated, for all *t*. In this case study, *T* ranges up to 16384.

The above setting is mapped to an SSM as follows:

• The logit-probability that the base *t* is methylated, i.e. $logit(p_{t})$ is the hidden state $X_{t}$ of the SSM. Therefore, $X_{1:T}=logit(p_{1:T})$.
• The number of successful tests at base *t* of the DNA sequence, for all four replicates ($y_{1:4,t}$) is the observation $y_{t}$ of the SSM, i.e. $y_{1:T}=y_{1:4,t}$. The number of tests at position *t* ($n_{1:4,t}$) is also part of the observations.

The transition, prior and observation densities of the SSM are constructed as follows to simulate the real setting: The transition density (2) for the single-tissue DNA case models the fact that the probabilities of methylation are dependent between subsequent DNA bases:

(18) $$\begin{array}{c} X_{t}∼Normal(X_{t-1},\tau_{t}^{2}),\;\;t>1 \end{array}$$

where $\tau_{t}^{2}$ is the transition variance. This is a function of a common variance $\sigma_{1}^{2}$ and the DNA physical position ($\delta_{t}$): $\tau_{t}^{2}=\sigma_{1}^{2}|\delta_{t}-\delta_{t-1}|$. The variance $\sigma_{1}^{2}$ represents the amount of dependence between the methylation states of subsequent bases.

The transition density in the multi-tissue DNA case (with two tissues) is similar but in this case a mixture of Gaussian densities is used instead of one Gaussian. This mixture represents the fact that two tissues with different inter-base dependence exist in the DNA sample:

(19) $$\begin{array}{c} X_{t}∼\sum_{c=1}^{2}[w_{c}\cdot Normal(X_{t-1},\psi_{tc}^{2})],\;\;t>1 \end{array}$$

where $w_{c}=0.5$ is the weight of the mixture component with index c∈{1,2} and $\psi_{tc}^{2}=\sigma_{c}^{2}|\delta_{t}-\delta_{t-1}|$. Here, $\sigma_{c}^{2}$ is the transition variance of component *c* (two variances $\sigma_{1}^{2}$ and $\sigma_{2}^{2}$ in total). The prior state equation (1) is $X_{1}∼Normal(0,1)$ in both the single- and multi-cases.

For the observation equations (3), a binomial model is used. This is suitable for modeling numbers of successes in a fixed number of tests. The equations are as follows:

(20) $$\begin{array}{c} y_{kt}∼Binomial(n_{kt},p_{kt}),\;k\in \{1,...4\},\;t>0 \end{array}$$

This means that the number of successful tests $y_{kt}$ at position *t* for replicate *k*, the number of tests $n_{kt}$ and the probability of methylation $p_{kt}$ are related according to a binomial likelihood. These observation equations apply to both case studies.

The only remaining part of the model is a connection between the logit-probabilities $logit(p_{1:T})=X_{1:T}$ that appear in the transition density (which are common for all replicates) and the probabilities $p_{1:4,1:T}$ that appear in the observation equations (one probability per replicate). This connection is achieved by the following equation:

(21) $$\begin{array}{c} logit(p_{kt})∼Normal(X_{t},\beta^{2}) \end{array}$$

where $\beta^{2}$ is the variance between replicates. Equation (21) is a random effect which models the diversity between replicates. It “bridges” the transition and observation equations.

Based on the above models, the two data sets (single- and multi-tissue) are generated using the following known parameter values: The DNA physical positions $\delta_{1:T}$ are uniform random integers in the range [1,100]. The number of tests $n_{kt}$ are uniform random integers in the range [1,50]. The probabilities of methylation are generated using the transition and prior equations of the SSM.

SSM inference (using pMCMC or ppMCMC)aims at estimating these probabilities (or rather the logit-probabilities), which are the hidden state of the SSM, as well as the vector of unknown parameters ***θ***. This vector is different in the two case studies:

• Single-tissue case study: The vector ***θ*** is $\theta =(\sigma_{1}^{2},\beta^{2})$ (dimension 2). When simulating the data, the vector is set to θ=(0.2,0.02)
• Multi-tissue case study: The vector ***θ*** is $\theta =(\sigma_{1}^{2},\sigma_{2}^{2},\beta^{2})$ (dimension=3). When simulating the data, the vector is set to $\left(\sigma_{1}^{2},\sigma_{2}^{2},\beta^{2})=(0.2,10.0,0.02\right)$.

To perform Bayesian inference, a Gamma Bayesian prior is used for each of the components of the ***θ*** vector. The parameters of the Gamma for the single-tissue case study are (1.2,100.0) for parameter $\sigma_{1}^{2}$ and (1.0,100) for parameter $\beta^{2}$. The parameters of the Gamma for the multi-tissue case study are (1.2,100.0) for parameters $\sigma_{1}^{2}$ and $\sigma_{2}^{2}$ and (1,100) for parameter $\beta^{2}$.

Based on the true values of the parameters which are used during simulation, it is clear that the single-tissue posterior admits one mode around $\left(\sigma_{1}^{2},\beta^{2})=(0.2,0.02\right)$. The multi-tissue posterior admits 2 modes, one in $\left(\sigma_{1}^{2},\sigma_{2}^{2},\beta^{2})=(0.2,10.0,0.02\right)$ and one in $\left(\sigma_{1}^{2},\sigma_{2}^{2},\beta^{2})=(10.0,0.2,0.02\right)$. This happens because the two mixture components of equation (19) have equal weights, and therefore either component can have mean 0.2 or mean 10.0, i.e. the posterior parameters $\sigma_{1}^{2}$ and $\sigma_{2}^{2}$ are interchangeable. The multi-modality of the posterior makes its exploration by pMCMC challenging, as will be shown in Section 7.

It is also worth noting that, since the data sets are generated using known parameters and SSM states, the posterior (4) is known for any given ***θ***. This fact can be used to assess the quality of pMCMC and ppMCMC samples, as will be shown in Section 7.3.

### 6.1 pMCMC and pMCMC tuning parameters

This section provides information on the choice of the various pMCMC and ppMCMC tuning parameters when sampling from the SSM case studies presented above.

**Number of samples and burn-in:** In all of the experiments of Section 7, the number of samples taken from the posterior is $N_{all}=11000$. Out of these, N=10000 are kept and BI=1000 are discarded as burn-in. This burn-in size is enough for convergence in all cases. In the evaluation section, N=10000 is considered as the sample size.

**ppMCMC temperatures:** The number of chains in the ppMCMC runs of Section 7 ranges from M=1 to M=5. An additive temperature progression was used, where $T_{1}=1$ and $T_{i+1}=Ti+2.5$.

**Proposal distributions:** As mentioned in Sections 2.3 and 4.1, multivariate Gaussian proposal distributions (with zero mean) are used in both pMCMC and ppMCMC. The covariance matrices of the proposals were tuned manually, by trying many candidate matrices. This tuning was done separately for pMCMC (for the single- and multi-tissue case studies) and for ppMCMC (for the multi-tissue case study). Only diagonal covariance matrices were considered.

In more detail:

• pMCMC: In the single-tissue case study (where the dimension of ***θ***) is 2, the covariance matrix used in all the experiments of Section 7 is $\Sigma_{s}=\left[\begin{array}{cc} 0.15 & 0.0\\ 0.0 & 0.008 \end{array}\right]$. In the multi-tissue case study (where the dimension of ***θ***) is 3, it is $\Sigma_{m}=\left[\begin{array}{ccc} 1.1 & 0.0 & 0.0\\ 0.0 & 1.1 & 0.0\\ 0.0 & 0.0 & 0.008 \end{array}\right]$.
• ppMCMC: Different covariance matrices are used for each chain of pMCMC. It was observed experimentally that larger chain temperatures require larger variances to be sampled efficiently, which is expected (a larger temperature leads to a smoother, more uniform distribution, which is explored more efficiently when a high-variance proposal is employed). In the multi-tissue case study, the following matrices were used in chains 1 to 5 respectively: $\Sigma_{m}1=\left[\begin{array}{ccc} 1.1 & 0.0 & 0.0\\ 0.0 & 1.1 & 0.0\\ 0.0 & 0.0 & 0.008 \end{array}\right]$, $\Sigma_{m}2=\left[\begin{array}{ccc} 2.0 & 0.0 & 0.0\\ 0.0 & 2.0 & 0.0\\ 0.0 & 0.0 & 0.015 \end{array}\right]$, $\Sigma_{m}3=\left[\begin{array}{ccc} 3.9 & 0.0 & 0.0\\ 0.0 & 3.9 & 0.0\\ 0.0 & 0.0 & 0.025 \end{array}\right]$, $\Sigma_{m}4=\left[\begin{array}{ccc} 5.8 & 0.0 & 0.0\\ 0.0 & 5.8 & 0.0\\ 0.0 & 0.0 & 0.030 \end{array}\right]$, $\Sigma_{m}5=\left[\begin{array}{ccc} 9.0 & 0.0 & 0.0\\ 0.0 & 9.0 & 0.0\\ 0.0 & 0.0 & 0.05 \end{array}\right]$

## 7 Investigation and results

This section evaluates the performance of the proposed ppMCMC algorithm and the pMCMC and ppMCMC FPGA architectures in two ways. The first way (Sections 7.2 and 7.4) is by comparing the FPGA accelerators for pMCMC and ppMCMC to state-of-the-art, optimized pMCMC implementations on a multi-core CPU and a GPU, generated by LibBi [10] (using single precision). Both the uni-modal and multi-modal SSM of Section 6 were defined in LibBi using the framework's domain-specific modeling language. The part of the evaluation (Section 7.3) compares the newly proposed ppMCMC algorithm to the pMCMC algorithm. Libbi is not used here because it is impossible to express ppMCMC using the LibBi language. Instead, sequential Matlab implementations of pMCMC and ppMCMC were used. This second comparison is not interested in parallelism; the goal is to compare the algorithms when no hardware acceleration is applied.

The two FPGA architectures were implemented in Vivado HLS 2014.1 using single precision floating point arithmetic. The PROTOIP framework [26] was used for prototyping. The architectures ran on a Xilinx ZC706 Zynq board (contains a Z-7045 FPGA). They were connected to a host PC (Core i7-2600, 16 GB RAM) through Ethernet. The FPGA clock was set to 144 MHz.

The LibBi CPU sampler ran on an Intel Core 2 Q9550 (2.83 GHz) with 8 GBs of RAM, using the Intel C++ compiler 2011, OpenMP multithreading (4 threads) and SSE vector parallelism (all provided by LibBi). The LibBi GPU sampler ran on an Nvidia Tesla C2050 hosted by an Intel Core 2 Q9550 CPU (2.83 GHz) with 8 GBs of RAM, using CUDA acceleration (again, provided by LibBi). Matlab code ran on the same machine used for the CPU sampler, without any exploitation of parallelism.

### 7.1 Resource utilization

Table 1 contains the FPGA's post-place and route resource utilization for the pMCMC and ppMCMC systems. The examined resources are Look-Up Tables (LUTs), Flip-Flops (FFs), Digital Signal Processing modules (DSPs) and Block RAM memories (BRAMs). The first three are computational resources, i.e. they are used to implement the computations of the algorithms, while the last is a memory resource, i.e. on-chip memory. ppMCMC takes up slightly more LUTs, FFs and DSPs due to the Exchange module. The BRAM utilization of ppMCMC is significantly larger due to the separate memory replicates for each chain. The critical computational resource (i.e. the one that limits *B*) for both samplers is the number of DSPs. The device's BRAMs force an upper limit on addressable problem sizes in pMCMC/ppMCMC ($T_{max}$ and $P_{max}$) and on *M* in ppMCMC ($M_{m}ax$), e.g. with a Z-7045 it is possible to use ($T_{max}=16384$, $P_{max}=16384$) or ($T_{max}=8192$, $P_{max}=32768$).

### 7.2 pMCMC: hardware comparison

The single-tissue case study of Section 6 was used to compare pMCMC accelerators. The performance metric used in this and the next sections is the Effective Samples per second that the sampler generates:

(22) $$\begin{array}{c} ES/\sec=\frac{ESS_{N}}{Time_{N}} \end{array}$$

where $Time_{N}$ is the wall clock time needed to generate *N* MCMC samples and $ESS_{N}$ is the effective sample size of the *N* MCMC samples (after removing burn-in). Effective sample size [27], [1] is the most common metric of MCMC mixing in the literature; it estimates how many independent (effective) samples the dependent MCMC samples are equivalent to, i.e. it quantifies the samples' “exploration value”. This is necessary for properly assessing pMCMC and ppMCMC performance, since both the number of particles (*P*) and the number of chains (*M*) influence mixing; a sample from pMCMC with P=512 has a different “exploration value” compared to a sample from pMCMC with P=256 or a sample from ppMCMC with M=2 and P=512. $ESS_{N}$ is estimated using the pMCMC/ppMCMC samples' autocorrelations (after removing burn-in) [27]:

(23) $$\begin{array}{c} ESS_{N}=\frac{N}{1+2\sum_{k=1}^{N}\alpha (k)} \end{array}$$

where α(k) is the autocorrelation at lag *k*. The summation in (23) is truncated when ρ(k) drops below 0.1. This is a common practice to reduce the variance of the estimator [27], [28]. The $ESS_{N}$ values shown below correspond to the first dimension of the sampled variable ***θ***, although the other dimensions behave in a similar way. By combining $ESS_{N}$ and $Time_{N}$, the ES/sec metric simultaneously considers mixing speed and raw sampling speed; it is the metric that ultimately interests a practitioner.

**Fig. 6:**
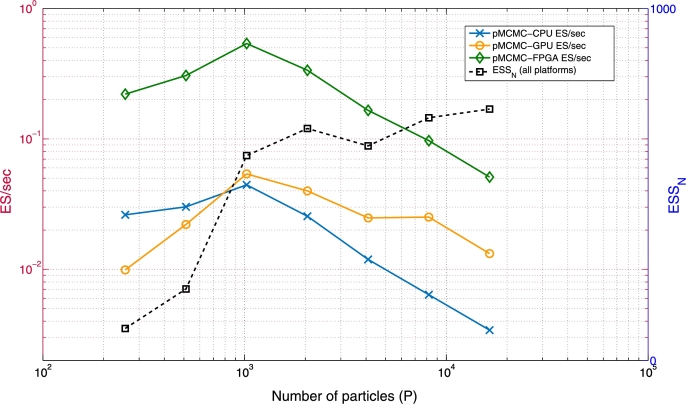
pMCMC: *ESS*_*N*_ and *ES*/sec of pMCMC sampler on multi-core CPU [10], GPU [10] and FPGA for varying *P* (single-tissue SSM, *T* = 1000, *N* = 10000). Runtimes include data transfers between the devices and the hosts.

Fig. 6 shows the ES/sec of the multi-core CPU, GPU and FPGA pMCMC samplers for different *P* when T=1000 and N=10000. $ESS_{N}$ (which is independent of the platform) is also shown. Both the ES/sec and $ESS_{N}$ values are averages over 10 independent pMCMC runs (for each *P*). $ESS_{N}$ increases rapidly for 256≤P≤1024 but slowly for larger *P* (which indicates that the likelihood estimate is already very accurate with P=1024). The CPU's $Time_{N}$ (not shown) increases proportionately to *P* because even 256 particles are enough to fully utilize the device. This leads to a CPU ES/sec which peaks at P=1024 and then drops, since $ESS_{N}$ grows faster than $Time_{N}$ for P≤1024 and slower than $Time_{N}$ for P>1024. The GPU's ES/sec also peaks at P=1024; it then drops at a slower rate than the CPU's ES/sec, since the GPU's massive parallel resources allow it to increase *P* without paying a large penalty in $Time_{N}$ ($Time_{N}$ grows slower than proportionately to *P*). The FPGA's performance also peaks at P=1024, achieving a ES/sec which is 12.1x and 10.1x higher than the peak CPU and GPU ES/sec respectively. The reduction of FPGA ES/sec for large *P* happens at a faster rate than in the GPU because the FPGA achieves almost full resource utilization earlier than the GPU (due to its highly optimized datapath); thus the FPGA's runtime grows almost linearly after P=1024. Finally, for P≥8192 the performance of all devices drops at a similar rate, since all of them are fully utilized (and thus runtime grows proportionately to *P*).

The above results demonstrate that a large number of particles is not always preferable for high sampling efficiency (when mixing is taken into account). For the same *P*, the FPGA is 8.4x–15.2x faster than the CPU and 3.8x–22.3x faster than the GPU. These latter speedups do not consider mixing, since mixing only depends on *P*. These speedups are useful for practitioners who have already tuned *P* in their pMCMC implementations. Similar results are observed when a different *T* is used (e.g. T=16000), i.e. the number of states does not affect performance (which is expected, since states are processed sequentially).

**Fig. 7:**
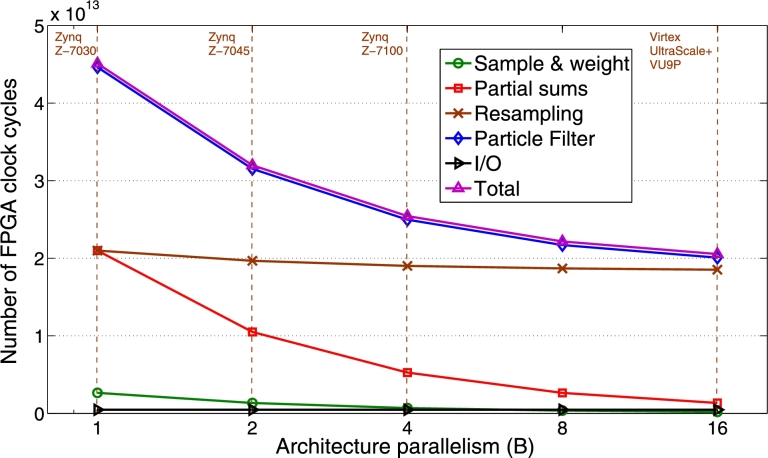
pMCMC: Cycles consumed by PF/pMCMC steps when architecture parallelism (*B*) varies (*T* = 1000, *P* = 16384, *N* = 10000, *T*_*max*_ = 16384, *P*_*max*_ = 16384). Four FPGA devices are shown (vertical lines) to demonstrate what *B* is achievable given each device's resources. Z-7045 was placed based on actual results. The other devices were placed based on projections.

The performance models of pMCMC (Section 5.4) were successfully validated using the above real runs (relative errors ranged from 0.7% to 7.5%). The models were then used to find how many cycles are spent at every step of the pMCMC architecture. Fig. 7 shows how the total cycles for a pMCMC run break down into the three PF steps and the cycles for I/O. It also shows how the cycles change when architecture parallelism (*B*), i.e. the number of parallel modules in each PF stage, increases (for T=1000 and P=16384). It is clear that the PF takes almost all of the runtime of pMCMC. Also, resampling becomes the bottleneck computation as *B* increases (46.5% of total cycles for B=1, 90.1% for B=16), since its cycles decrease only slightly when instantiating more parallel modules. This is due to the particle replication step ($P\cdot Lat_{rep}$ in (14)), which is the only non-constant term in (12)–(14) which does not have a denominator *B* (since replication cannot be parallelized). Other stages' cycles decrease almost proportionately to *B*. Total cycles decrease with larger *B* but speedup gains are diminishing. I/O cycles come from real runs.

### 7.3 ppMCMC vs. pMCMC: algorithm comparison (Matlab)

In this section, the performance of the proposed ppMCMC algorithm is compared to pMCMC when sampling from the multi-tissue posterior distribution, focusing on the algorithmic strengths of each approach (and ignoring the gains from parallelizing the computations).

**Fig. 8:**
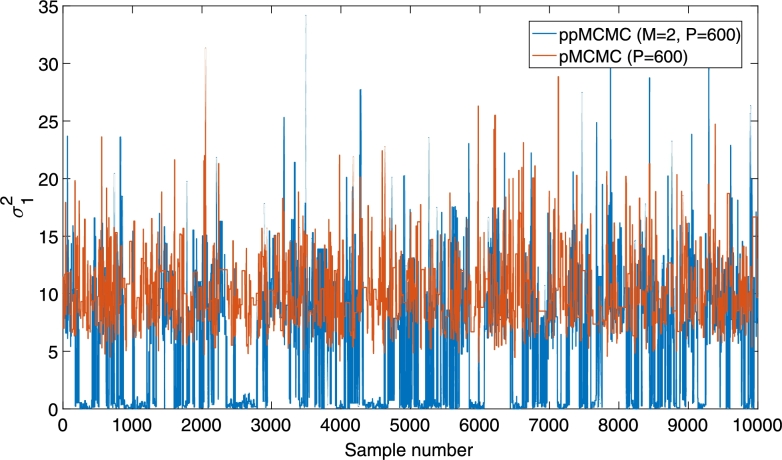
Sample trace of pMCMC (*P* = 600) and ppMCMC (*M* = 2, *P* = 600) for the parameter $\sigma_{1}^{2}$ of the multi-tissue SSM posterior (*T* = 200). Both samplers are implemented in Matlab. Burn-in samples have been removed.

Fig. 8 shows the sample trace of the pMCMC and ppMCMC samplers (with M=2 chains) when targeting the multi-tissue SSM posterior of Section 6. Only the first dimension of the *θ* vector, corresponding to the $\sigma_{1}^{2}$ parameter, is shown. The posterior has two modes, one around $\sigma_{1}^{2}=0.2$ and one around $\sigma_{1}^{2}=10.0$ (for details see Section 6). It is clear that pMCMC discovers only the second mode (around $\sigma_{1}^{2}=10.0$) and it cannot escape from it. In contrast, ppMCMC (with just two chains) jumps between the two modes frequently.

**Fig. 9:**
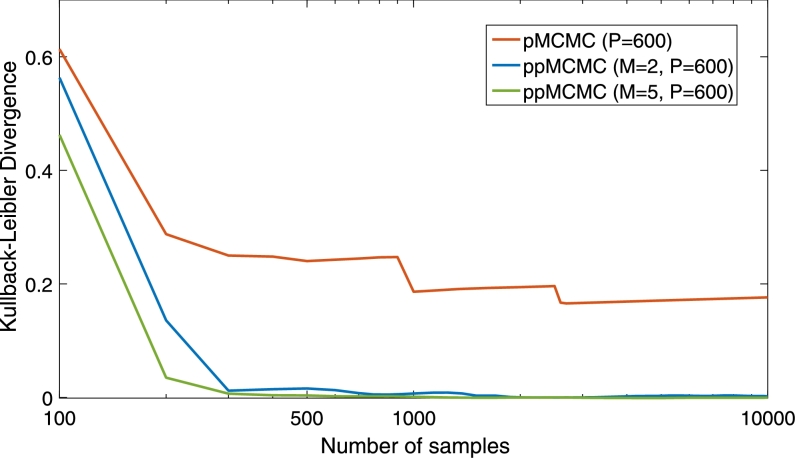
KLD between the true posterior and the posterior estimates of pMCMC and ppMCMC samples. The estimates are constructed using a kernel density estimator. Curves for pMCMC (*P* = 600) and ppMCMC (*M* = 2, *P* = 600 and *M* = 5, *P* = 600) are shown. Both samplers are implemented in Matlab. Burn-in samples have been removed.

Fig. 9 demonstrates the suitability of ppMCMC for sampling multi-modal posteriors even more clearly. For the same posterior as above, the figure shows the Kullback–Leibler divergence (KLD) [29] between the true SSM posterior and the estimate of the SSM posterior, constructed using the pMCMC or ppMCMC samples. KLD is a metric that quantifies the discrepancy between two probability densities and is computed as follows:

(24) $$\begin{array}{c} KLD=\int p(x)log(\frac{p(x)}{q(x)})dx \end{array}$$

where p(x) is the “true” density and q(x) is the approximation. KLD has the property KLD≥0.0. Its value decreases as the two densities become more similar and KLD=0 only when the densities are identical. Here, p(x) is the SSM posterior (see (4)) and q(x) is the posterior sample estimate, which is constructed by applying a kernel density estimator to the pMCMC or ppMCMC samples. The kernel of the estimator is Gaussian kernel and the bandwidth is set to BW=0.85. The figure shows that the KLD of pMCMC decreases at a slow rate and does not manage to converge to zero, even when N=10000 samples have been generated. This happens because pMCMC gets stuck in one of the two modes (as previously shown in Fig. 8) and thus its samples cannot represent the posterior adequately. In contrast, the KLD of both ppMCMC versions converges to zero, showing that they explore the whole posterior. Also, the M=5 version converges faster, showing the benefit from using more chains.

**Fig. 10:**
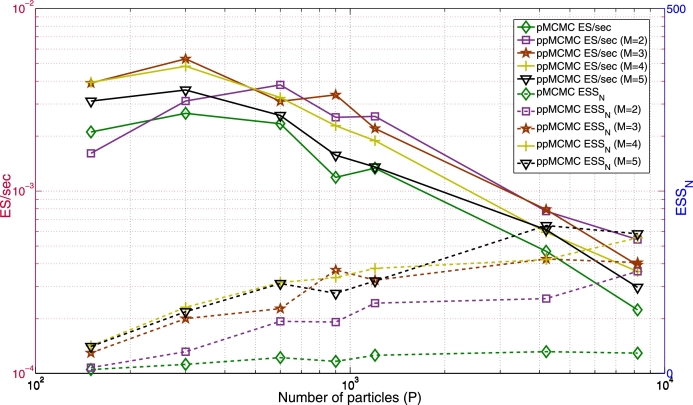
*ESS*_*N*_ and *ES*/sec of pMCMC and ppMCMC in Matlab when *M* and *P* vary (multi-tissue SSM, *T* = 200 and *N* = 10000).

Fig. 10 shows the $ESS_{N}$ and ES/sec of the Matlab implementations of pMCMC and ppMCMC (M=2 to M=5) when *P* ranges from 150 to 8200, T=200 and N=10000. Both the ES/sec and $ESS_{N}$ values are averages over 10 independent pMCMC runs (for each *P* and each *M*).

Larger *M* (for ppMCMC) and *P* (for both methods) increase $ESS_{N}$ (although there are some fluctuations due to the $ESS_{N}$ estimator) but they also increase $Time_{N}$ (not shown but increases proportionately to *M* and *P*). The ES/sec that results from this trade-off varies. In pMCMC it is maximized for P=300, while in ppMCMC it is maximized for (P=300, M=3). The latter is 1.96x faster than the former. For the same *P*, using multiple chains is more efficient than single-chain pMCMC by up to 2.8x (despite the extra computational cost). The only ppMCMC case which is slower than pMCMC is (P=150, M=2). The figure also shows that increasing *P* above some number decreases ES/sec for any *M*. This is because the gains in $ESS_{N}$ stop increasing for large *P*, while $Time_{N}$ increases proportionately to *P*. Optimal ES/sec values are achieved for P=300 or P=600 (depending on *M*).

### 7.4 ppMCMC vs. pMCMC: hardware comparison

**Fig. 11:**
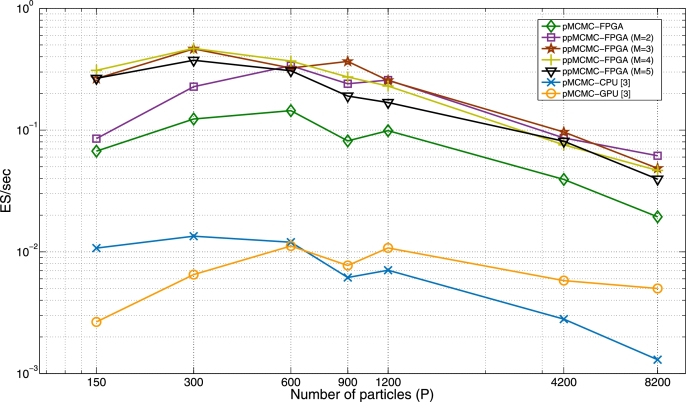
*ES*/sec of FPGA ppMCMC, FPGA pMCMC, CPU pMCMC and GPU pMCMC for varying *P* (multi-tissue SSM, *T* = 200, *N* = 10000).

Fig. 11 compares the ES/sec of the pMCMC and ppMCMC FPGA architectures to the ES/sec of the CPU and GPU pMCMC implementations in LibBi (using the multi-tissue SSM with T=200, N=10000). Both the ES/sec and $ESS_{N}$ values are averages over 10 independent pMCMC runs (for each *P* and each *M*). The ES/sec of the CPU pMCMC peaks for P=300 and drops for larger *P* because the extra computational cost outweighs the $ESS_{N}$ benefit (the latter is equal to the $ESS_{N}$ in Fig. 10). The ES/sec of the GPU pMCMC peaks at P=600 and remains close to its peak value for larger *P* because the computational cost here is smaller than in the CPU case (the GPU has more parallel resources to utilize and pays small $Time_{N}$ penalties for larger *P*). The ES/sec of the FPGA pMCMC peaks at P=600. This peak performance is 10.7x and 12.8x higher than the peak CPU and GPU performances respectively. The fastest ppMCMC configuration (P=300, M=4) is 34.9x, 41.8x and 3.24x faster than the fastest CPU, GPU and FPGA pMCMC configurations. Notice that the optimal ppMCMC configuration changes from Matlab to the FPGA implementation. By comparing Fig. 10, Fig. 11, it can be observed that combinations with large *P* and (especially) *M* are more “favoured” in the FPGA than they are in Matlab. This happens because 1) FPGA runtime does not increase proportionately to *P* and *M* as in Matlab (it increases at a slower rate) and 2) chain pipelining in the ppMCMC architecture improves efficiency for M>1. For constant *P*, adding chains improves ES/sec by up to 3.96x vs. pMCMC (2.8x in Matlab). These results confirm that the combination of ppMCMC with a specialized architecture offers significant gains over existing algorithms and accelerators when the posterior is multi-modal.

These results reveal that ppMCMC and its FPGA architecture offer large gains in performance compared to other algorithms and devices when the target distribution is multi-modal.

### 7.5 Power efficiency

This section compares the power efficiency of the CPU, GPU and FPGA implementations of pMCMC and ppMCMC. The metric used is the number of Effective Samples that can be generated per Joule of energy consumed (*ES*/J). The power consumption of the FPGA is estimated using the Xilinx Power Estimator [30], assuming maximum resource utilization. The estimate is $PW_{FPGA}=19.2\;W$. The CPU and GPU power consumption is taken to be equal to the nominal values given by the manufacturers ($PW_{CPU}=95\;W$ and $PW_{GPU}=238\;W$ respectively [31], [32]).

Table 2, Table 3 show the best *ES*/J achieved by each algorithm-device combination (after trying all possible parameter values for *P* and *M*), for the two case studies of Section 6. The values in the parentheses are *ES*/J speedups vs. the CPU implementation for the same case study. The FPGA implementation of pMCMC generates up to 60.1x more effective samples per Joule compared to CPU and GPU implementations which use LibBi. For the FPGA implementation of ppMCMC, the speedup reaches 173x. Also, the ppMCMC FPGA sampler is 3.25x more power efficient than the pMCMC FPGA sampler.

These numbers show that the FPGA has a significant power efficiency advantage, which can prove critical for SSM applications where energy consumption is a major concern. These include large-scale SSM inference on data centres (e.g. for genetic or financial applications), as well as SSM inference for embedded tasks (e.g. robot localization). In the former, electricity bills are the main operational cost. In the latter, the power budget is limited because embedded platforms run on batteries. Therefore, the adoption of FPGAs for SSM inference can either drive cost savings or allow much more complex analyses to be performed on the same power budget.

## 8 Conclusions and future work

This work introduced ppMCMC, an MCMC algorithm which combined pMCMC with population-based MCMC to improve mixing when sampling from multi-modal SSM posteriors. FPGA architectures tailored for pMCMC and ppMCMC were proposed, taking advantage of the parallelism within the algorithms to increase sampling efficiency. By exploiting the design space trade-offs of the hardware architectures (which include the number of particles and the number of chains), it was shown that the parameter combination which maximizes performance can be found. The results revealed that the new algorithm and FPGA samplers increase sampling efficiency significantly (by up to 1.96x and 41.8x respectively) compared to state-of-the-art CPU and GPU accelerators, bringing large-scale SSM inference within reach.

Future work will focus on: 1) Utilizing distributed resampling methods to lift the particle replication bottleneck (which prevents the sampling speed to increase proportionately to the FPGA size), 2) Accelerating SMC^2^, a promising competitor of pMCMC for SSM inference.

## Figures and Tables

**Algorithm 1:**
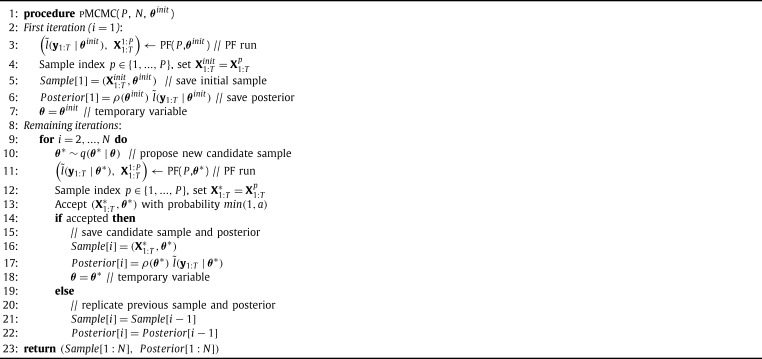
Particle MCMC.

**Algorithm 2:**
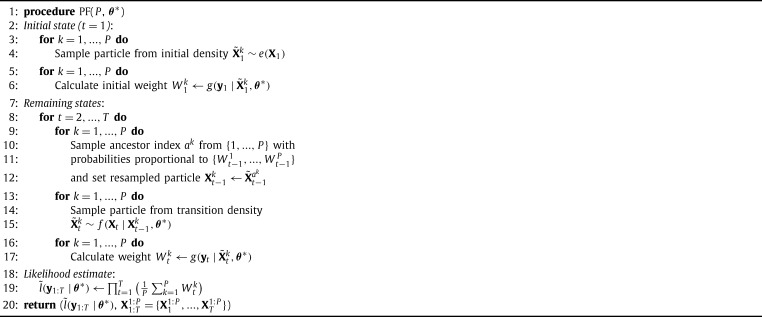
Bootstrap Particle Filter.

**Algorithm 3:**
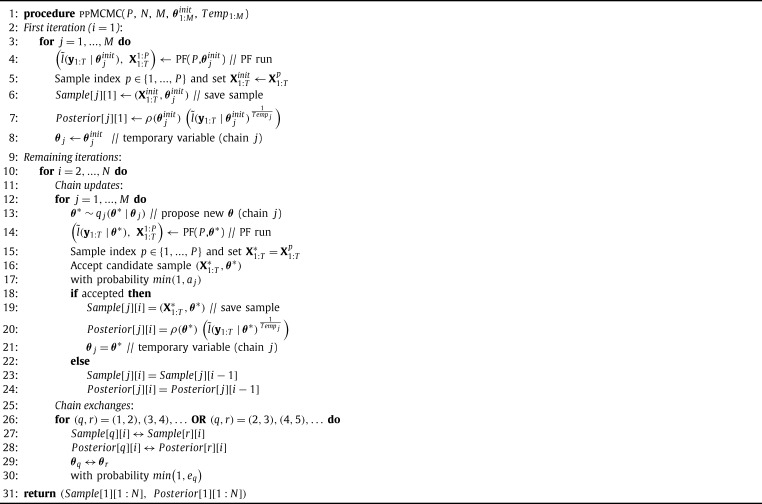
Population-based Particle MCMC.

**Table 1:** Resource utilization of pMCMC/ppMCMC. *B* = 2, *P*_*max*_ = 8192, *T*_*max*_ = 8192, *M*_*max*_ = 5 (ppMCMC only).

Block name	LUTs	FFs	DSPs	BRAMs
pMCMC block	85593	109017	710	223
ppMCMC block	87122	110543	752	496
Others (CPU, DMA)	729	926	0	0
pMCMC total	86322	109943	710	223
ppMCMC total	87851	111469	752	496

Z-7045 resources	218600	437200	900	545

**Table 2:** Power efficiency – single-tissue SSM.

Algorithm	CPU	GPU	FPGA
pMCMC	4.67 ⋅ 10^−4^ (1x)	2.26 ⋅ 10^−4^ (0.48x)	2.81 ⋅ 10^−2^ (60.1x)

**Table 3:** Power efficiency – multi-tissue SSM.

Algorithm	CPU	GPU	FPGA
pMCMC	1.41 ⋅ 10^−4^ (1x)	4.69 ⋅ 10^−5^ (0.33x)	7.50 ⋅ 10^−3^ (53.1)
ppMCMC	–	–	2.44 ⋅ 10^−2^ (173.0x)
